# Achieving the Minimum Roughness of Laser Milled Micro-Impressions on Ti 6Al 4V, Inconel 718, and Duralumin

**DOI:** 10.3390/ma13204523

**Published:** 2020-10-12

**Authors:** Naveed Ahmed, Ateekh Ur Rehman, Kashif Ishfaq, Rakhshanda Naveed, Khaja Moiduddin, Usama Umer, Adham E Ragab, Ayoub Al-Zabidi

**Affiliations:** 1 Department of Industrial Engineering, College of Engineering and Architecture, Al Yamamah University, Riyadh 11512, Saudi Arabia; naveed527@gmail.com; 2 Department of Industrial Engineering, College of Engineering, King Saud University, Riyadh 11421, Saudi Arabia; aragab@ksu.edu.sa (A.E.R.); 439106932@student.ksu.edu.sa (A.A.-Z.); 3 Department of Industrial and Manufacturing Engineering, University of Engineering & Technology, Lahore 54890, Pakistan; kashif.ishfaq@uet.edu.pk (K.I.); rakhshanda@uet.edu.pk (R.N.); 4 Advance Manufacturing Institute, College of Engineering, King Saud University, Riyadh 11421, Saudi Arabia; khussain1@ksu.edu.sa (K.M.); uumer@ksu.edu.sa (U.U.)

**Keywords:** micro-impressions, laser milling, titanium alloy, nickel alloy, aluminum alloy, surface roughness, parametric optimization, statistical modeling

## Abstract

Titanium-aluminium-vanadium (Ti 6Al 4V) alloys, nickel alloys (Inconel 718), and duraluminum alloys (AA 2000 series) are widely used materials in numerous engineering applications wherein machined features are required to having good surface finish. In this research, micro-impressions of 12 µm depth are milled on these materials though laser milling. Response surface methodology based design of experiment is followed resulting in 54 experiments per work material. Five laser parameters are considered naming lamp current intensity (*I*), pulse frequency (*f*), scanning speed (*V*), layer thickness (*LT*), and track displacement (*TD*). Process performance is evaluated and compared in terms of surface roughness through several statistical and microscopic analysis. The significance, strength, and direction of each of the five laser parametric effects are deeply investigated for the said alloys. Optimized laser parameters are proposed to achieve minimum surface roughness. For the optimized combination of laser parameters to achieve minimum surface roughness (Ra) in the titanium alloy, the said alloy consists of *I* = 85%, *f* = 20 kHz, *V* = 250 mm/s, *TD* = 11 µm, and *LT* = 3 µm. Similarly, optimized parameters for nickel alloy are as follows: *I* = 85%, *f* = 20 kHz, *V* = 256 mm/s, *TD* = 8 µm, and *LT* = 1 µm. Minimum roughness (Ra) on the surface of aluminum alloys can be achieved under the following optimized parameters: *I* = 75%, *f* = 20 kHz, *V* = 200 mm/s, *TD* = 12 µm, and *LT* = 3 µm. Micro-impressions produced under optimized parameters have surface roughness of 0.56 µm, 2.46 µm, and 0.54 µm on titanium alloy, nickel alloy, and duralumin, respectively. Some engineering applications need to have high surface roughness (e.g., in case of biomedical implants) or some desired level of roughness. Therefore, validated statistical models are presented to estimate the desired level of roughness against any laser parametric settings.

## 1. Introduction

Titanium alloy (Ti 6Al 4V), nickel alloy (Inconel 718), and aluminum alloy (AA 2024) are widely used materials in various engineering applications such as biomedical implants, aerospace, ship building, automotive, and many more as reported by Lupi et al. [1], Nalli et al. [2], and Wang et al. [3]. The production of these materials in the form of near-net-shape products is extensively carried out through several means, especially selective laser melting (SLM) as adopted by Fan and Feng [4], and electron beam melting (EBM) used by Moiduddin et al. [5]. The surface roughness of these produced alloys through the said processes is experienced to be very high. For example, according to research conducted by Sidambe [6], electron beam melting allows for obtaining a range of surface roughness R_a_ values between 15.8 µm and 54.3 µm which are considered as considerably high. In another study presented by Anwar et al. [7], a surface roughness of additively manufactured parts is reported as R_a_ = 31 µm. These surface roughness values are very high since Klocke et al. [8] and Uddin [9] stated that the parts used in aerospace and biomedical applications have substantially low surface roughness. Post processing (machining or polishing) is frequently required to make the parts useable in end-use applications as of the aerospace and biomedical sector.

Titanium alloys, especially Ti 6Al 4V, have high chemical reactivity and low thermal conductivity due to which Ti 6Al 4V is considered as difficult-to-machine material as per the results of Abbas et al. [10]. Nickel alloys, especially Inconel 718, is also considered as difficult-to-cut material owing to have lower thermal conductivity, high work hardness, and high toughness. Machno et al. [11] reported that the presence of abrasive carbide particles in nickel alloys creates much more difficulty during conventional machining processes. Aluminum alloys are relatively less hard as compared to titanium and nickel alloys. It has been shown by Haddag et al. [12] that the difficulty experienced during conventional machining of aluminum alloys is the formation of build-up edge alongside the tool cutting edge. The adhesion and the fusion property of the aluminum alloys seriously affect the tool life and, as a result, the roughness of the machined surface is compromised. These are the findings of Rawangwong et al. [13]. For these reasons, machining of AA 2024 is also considered as challenging and it offers difficulty to maintain industrial tolerances in the machined parts dealt with a conventional means of machining [14]. Aluminum alloys are also considered as problematic even processed through laser machining. The reason behind this difficulty is reported by Dubey et al. [15] as thermo-physical properties. Another route to machine hard materials is the assisted machining where an additional source of energy is used to soften the localized area in front of the cutting tool. A very common type of energy assisted machining is the laser assisted machining as can be seen in a research conducted by Kim et al. [16]. In another study of Kim et al. [17], induction assisted milling of Inconel 718 has been reported and optimized process parameters are proposed. For the same analogy, plasma has been used as an assisting source of energy to machine Ti 6Al 4V, and the process is termed as plasma-assisted machining [18]. In this way, by the use of additional source of energy to soften the localized area ahead of the cutting tool, the cutting forces can be reduced and tool life can be enhanced by minimizing the frequency of tool failure.

Since the said alloys (titanium, nickel and aluminum alloys) are considered as difficult-to-machine, nonconventional machining processes are found to be more suitable to work on these materials. Nam et al. [19] said that the high tool wear rate is the main challenge while machining difficult-to-cut materials. Formation of circular micro-impressions on Ti 6Al 4V have been realized through electric discharge machining (EDM). Geometrical errors and surface roughness have been evaluated by employing different tool electrode materials. The suitable tool material and EDM parameters are suggested to produce micro-impressions with minimum surface roughness. The graphite tool allowed the highest surface roughness (R_a_ 8.85 µm), whereas aluminum electrode resulted in a minimum roughness (1.05 µm) of the micro-impressions produced by Ahmed et al. [20]. Similarly, micro-grooves have been produced in the titanium alloy as reported by Lei et al. [21] through EDM. Likewise, Rahul et al. [22] machined different grades of nickel alloy through EDM to explore the effect of process parameters over the surface roughness and tool wear rate. In another consulted research work of Misal et al. [23], surface roughness analysis of Inconel 718 has been carried out to understand the effect of photochemical machining parameters. Due to the difficulty in machining of Inconel 718, deep hole drilling has been realized through electric discharge drilling by Machno [24]. He studied the effect of process parameters over the quality of drilled holes. Kumar et al. [25] investigated parametric effects of water jet cutting on Inconel 718 to understand the machining behavior.

For the purpose of micro-machining, other non-conventional processes are also used, but they offer several limitations as well. Sivaprakasam et al. [26] machined Ti 6Al 4V through micro-WEDM and achieved a very low surface roughness (R_a_ = 0.789 µm). However, WEDM is not applicable to produce blind cavities. The presence of kerf width is another issue of the WEDM. As per the work presented by Huang et al. [27], micro-EDM milling can be another alternate to produce cavities or micro-impressions. However, high tool wear is the major problem of micro-EDM milling. Wearing also poses a dimensional inaccuracy issue. Radial overcut present in the machined feature due to inter-electrodes gap is another challenge of micro-EDM milling. Micro-electrochemical machining (micro-ECM) is another process used by Zeng et al. [28] capable of machining 3D micro-features with comparable surface roughness (R_a_ = 0.707 µm). However, the problem of electrode wear still remains challenging during ECM.

Laser machining is another viable route to process difficult-to-cut materials. The laser machining process, especially laser milling, involves various laser parameters. The important parameters reported in literature, for example by Büttner et al. [29], are laser intensity or power, scanning cycle, pulse repartition rate, pulse overlap, scan strategy, laser spot diameter, and number of laser passes to machine a particular feature. Several laser machining processes are in practice including laser drilling, milling, and cutting in which the mentioned laser parameters are needed to be tuned as per machining requirements. However, the appropriate setting of each parameter is not an easy task. If the optimized laser parameters are available for a particular type of job, the machinist feels as if they are working in a comfortable zone. Another categorical use of laser beam machining is to produce micro-texturing and surface modifications of a variety of substrates. In surface texturing, the role of roughness is considered as crucial. Various types of micro-textures, micro-cavities, micro-channels, and micro-arrays are the typical and unique applications of the laser milling process. These features are required in several types of engineering products that are directly used in mold making, biomedical, heat-exchanges, heat sink, non-stick coatings of molds, and other applications [30]. For example, Lee et al. [31] produced spherical and elliptical shaped 3D surface patterns by the use of laser micromachining and discussed the suitable process parameters to achieve the patterns. Similarly, Paula et al. [32] developed micro-pores in a nanofubrous membrane by the utilization of the femtosecond laser. The Nd:YVO4 pulsed laser can be used to produce micro-textures on Ti 6Al 4V, and AA 2024 was used by Ahuir-Torres et al. [33]. The researchers have investigated the effect of laser parameters over the quality of dimple shaped textures. Another study related to the formation of surface textures on AA 2024 through picosecond laser machining has been reported by the same researchers, Ahuir-Torres et al. [34], wherein three kind of textures are produced including dimple-shaped, concentric ring shaped, and a cross-groove type pattern.

Although the laser machining is very advantageous, it is very widely stated that the laser machining involved a complicated machining phenomenon, and the optimized laser parameters are essential to achieve desired machining results as perceived from the research findings of Schille et al. [35]. For example, as per the results of Dhaker et al. [36], the quality of laser drilled holes in Inconel 718 is not easy to achieve without the absence of optimized parameters. Williams et al. [37] studied the effect of fiber laser parameters during machining of aluminum. Kuar et al. [38] proposed an optimized set of Nd:YAG laser parameters to perform micro-machining of die-steel. They have used response surface methodology design of experiments and also developed regression models for achieving minimum height of recast layer and a maximum depth of groove of the micro-groove machined. Dubey et al. [15] investigated the effects of laser process parameters to cut an aluminum sheet through laser cutting. They have suggested the most appropriate settings of laser parameters to cut the sheet with optimal kerf quality. Likewise, Sharma and Yadava [39] proposed optimized values of Nd:YAG laser cutting parameters to achieve the straight profiled cut in aluminum with minimum kerf variation. Since many studies in the field of laser machining are reported on the development of mathematical models and optimized values of laser parameters.

Similarly, the development of statistical models helps the machinist to estimate the machining results in terms of desired response measures as can be found in research conducted by Hossain et al. [40]. Therefore, several researchers have proposed statistical models in the field of machining to predict the machining response prior to perform actual machining job. For example, statistical models for material removal of ultrasonic machining of titanium have been developed by Kumar et al. [41]. Yu et al. [42] investigated the laser texturing parametric effects on the bio-functionality of a titanium alloy. They have developed a correlation between laser parameters and the geometry of micro-grooves. Ghosal and Manna [43] investigated the effect of ytterbium laser parameters on material removal rate and tapering phenomenon during laser milling of an aluminum matrix composite. They have developed the statistical models and proposed optimized laser parameters to achieve maximum material removal rate with minimum taper angle. Wee et al. [44] performed ultraviolet laser micro-machining on mold inserts and investigated the dimensional performance (ablation depth) and machining quality. From the literature, it can be observed that each study is dedicated for a specific substrate material and for a certain machining feature. This is why it is stated by Mukherjee et al. [45] that searching for an optimized laser parameters is essential to achieve a desired level of machining performance in each particular material. Likewise, Hedieh et al. [46] investigated the effect of laser pulse overlapping over the machined surface quality. It has been stated that, for the quality machining results, it is better to have the right choice of laser parameters. They have produced micro-channels in polymers and studied their profiles.

In the present study, laser milling of three widely used industrial alloys (titanium alloy; Ti 6Al 4V, nickel alloy; Inconel 718, and aluminum alloy; AA 2024) has been carried out. Micro-cavities of 12 µm machining depth are produced in each alloy. Five laser parameters were taken as the variable to investigate their effect on the surface quality of the micro- cavities. Response surface methodology based design of experiment has been planned resulting in 54 experiments to be performed on each of the three alloys. The influences of laser parameters are evaluated through the study and analysis of parametric effects, interaction plots, and microscopy techniques. Analysis of variance, Pareto analysis, and normal plot analysis for standardized effects have been discussed to identify the significant parameters along with their strength and direction of effect. Moreover, optimized settings of laser parameters are proposed and verified to produce the micro-impressions with minimum surface roughness. However, many engineering applications need to have rough surfaces like texturing and biomedical implants. Finally, statistical models have been developed and carefully validated to estimate and to control the roughness of the milled cavities as per desired level. By the use of statistical models, the selection of suitable laser parameters can be easily made with the objective of achieving the laser milled surface with the desired level of surface roughness.

## 2. Materials and Methods

Titanium alloy (Ti 6Al 4V) [47], nickel alloy (Inconel 718) [48], and duralumin (AA 2024) [49] are well known materials used in biomedical, aerospace and other several applications. Laser milling of three aerospace materials is carried out in this investigation. Titanium alloy (Ti 6Al 4V), nickel alloy (Inconel 718), and duralumin (AA 2024) were the chosen work materials. Material removal during laser milling is a function of laser energy and substrate’s properties such as absorptivity, reflectivity against laser light, and melting point. Important properties of the substrate materials are shown in Table 1. Micro-impressions or cavities of 3 mm × 6 mm were milled with a milling depth of 12 µm. Q-switched Nd:YAG (DMGMORISEKI, Bielefeld, Germany) laser is used as the energy source for milling. The laser has a wavelength of 1064 nm with a spot size of 20 µm. Since laser milling involved several process parameters, after performing a long list of trial experiments, the five most influential process parameters are identified including lamp current intensity (*I*), pulse repetition rate (*f*), laser scan speed (*V*), layer thickness per laser scan (*LT*), and pulse overlap also called track displacement (*TD*). The three levels of each of these five variables are shown in Table 2. All of the other parameters like spot size, wave length, pulse duration (10 µs), and scanning mode (randomized scanning) were kept constant. The focus of the laser spot was set on the top surface of workpiece. Since the initial roughness (R_a_) of the work samples was around 6 µm, a milling depth of 12 µm was chosen so that the milled impressions totally have new surface after laser irradiations. It is also worth mentioning that the initial work surfaces should be dull and rough for the effective use of laser milling. The laser energy needs to be absorbed by the substrate surface. The smoother the initial surface, the greater the laser beam reflection. High reflection causes the loss of irradiated energy. Therefore, it is recommended that the initial surface roughness should be high in order to achieve maximum absorption of laser light. This is the reason that absorptivity of the substrate material is a well-reported factor to be considered during laser milling. An increase in surface roughness increases the absorptivity and effective surface area exposed to laser irradiation, research reported by Mustafa et al. [50]. Rough surfaces augment the light absorbance and maximize the energy utilization by minimizing the reflection. A similar fact regarding light absorbance can be found in a study conducted by Rudenko et al. [51] wherein nanobumps present on the initial substrate surface are favorable to maximize the absorption of laser irradiation. This is the reason that polished surfaces or reflective materials (such as silver, copper, gold, and aluminum), as stated by Naeem [52], are difficult to machine by laser machining. As per the consulted work of Miyamoto and Knorovsky [53], silver is one of the most difficult materials to laser process, requiring almost 20 times more power than is required to melt steel. Tian and Wang [54] also reported the similar observations.

**Figure 1:**
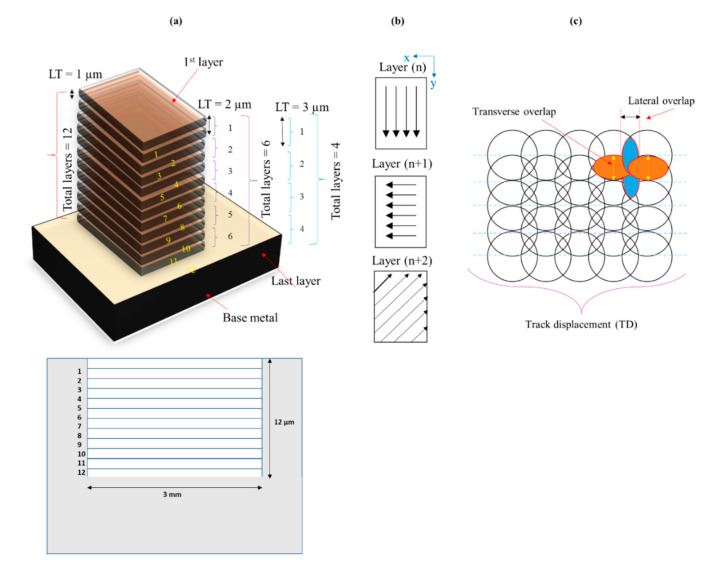
Schematics of laser milling phenomenon for micro-impressions; (**a**) number of layers against different layer thickness values, (**b**) mode of scanning cycle, and (**c**) pulse overlap in terms of track displacement.

Lamp current intensity is the primary parameter in laser machining. Some researchers, such as Liu et al. [67] and Li et al. [68], used the lamp power, whereas some studies have reported the lamp current intensity as a primary laser parameter such as Vincent et al. [69], Abdo et al. [70], and Mohammed et al. [71]. A laser source used in this study can deliver maximum power of 30 W by the utilization of 100% lamp current intensity. After trial experiments, three levels of current intensity were taken as the influential level including current intensity of 75%, 80%, and 85%. Similarly, three levels of pulse frequency (10, 15, 20 kHz) and scanning speed (200, 300, and 400 mm/s) were selected. Another important variable is the layer thickness (*LT*). It is a thickness of the expected layer to be removed after each scanning cycle completed on the exposed work surface. The focus of the laser spot is a function of input value of the layer thickness. For example, by setting 1 µm layer thickness, it is anticipated that the focus of the laser spot is at the top surface and, after revolving 1 µm layer from the work surface, the laser galvanometer adjusts its focus for the upcoming fresh layer. Likewise, 2 µm layer thickness allows the galvano head to adjust the laser focus by 2 µm after completing each scanning cycle. In this way, the focus stays the same with respect to the top surface. Thus, three levels of *LT* (1, 2, and 3 µm) have been selected to see the milling effect and to decide the most appropriate one. To finish the milling depth of 12 µm, scanning is carried out for 12 layers if *LT* is 1 µm. Similarly, using 2 µm layer thickness results in 6 layers (or the number of scanning cycles). This concept is schematically illustrated in Figure 1a. It must be noted that the laser scanning needs to follow a scan pattern. In this research, a randomized scanning pattern is followed. For any layer (nth layer), the scanning is carried out along the *y*-direction and, for the next layer (n + 1 layer), the scanning follows the *x*-direction and, for the subsequent layer (n + 2 layer), it moves along 45° to the *x*-direction as depicted in Figure 1b. This cycle continues until the desired milling depth is achieved. The overall pattern is called the randomized scanning pattern. The reason behind selecting the randomized pattern is to avoid the scanning marks on the milled surface as the problem of visible scanning marks was realized during trial experiments when unidirectional scan was used. Pulse overlap has also been found to be an imperative parameter during trials. During a scanning cycle, successive laser spots against each pulse overlap with each other. It generates two types of overlapping, called traverse overlap and lateral overlap, as shown in Figure 1c. Excessive overlapping of laser spots causes over-melting of the substrate surface, whereas very low overlapping generates visible milling marks affecting the roughness of the micro-cavities. Overlapping is also termed as track displacement (*TD*). Therefore, three levels of track displacement were taken (8, 10, and 12 µm) to investigate their effect.

The machining was carried out in a closed chamber. Usually, in laser machining, the removal of debris is carried out with the help of inert gas. However, in our experimental setup, the closed machining chamber was equipped with a suction mechanism that allows debris to be flushed away under the action of suction.

Based on the number of variables and their levels, response surface methodology (RSM) based design of experiment was planned. Among different designs of RSM, face centered cubic (FCC) was adopted resulting in 52 experiments. The same set of experiments was repeated for each of the work materials (Ti 6Al 4V, Inconel 718, and AA 2024). Thus, in addition to trial runs, a total 156 experiments were performed under design of experiments (DOE). The roughness (R_a_) of each sample was very carefully measured for three times using a surface roughness meter (Surtronic S-128, Taylor-Hobson, Leicester, UK). For the roughness measurement, an evaluation length of 4 mm was selected to get reliable results. Moreover, roughness from three different regions of each sample was recorded to get robust results. The average value of surface roughness of micro-impressions has been reported.

The microscopic analysis of the laser milled-surface was carried out. The effects of each of the five laser parameters over the roughness of the micro-cavities have been critically evaluated with the help of parametric effects, main effect plots, and interaction plots. Furthermore, analysis of variance is conducted to statistically identify the severity of parametric effects. Finally, optimized parameters are recognized to achieve the laser-milled micro- cavities with minimum possible surface roughness. The optimized parameters were ultimately verified by performing validation tests for each of the three work materials.

The geometry of the machined impressions or cavities was measured, especially the depth of cavity. The measurement was taken out with the help of a “depth measuring probe of lasertec 40”. The measurement resolution of the probe was 1 µm. Since the scope of the present work is focused on the surface roughness of the milled impressions, the analysis of machined depth has not been discussed.

## 3. Results and Discussion

After performing the laser milling experiments, each sample was examined and measurements of surface roughness were carried out. Since the data set is very extensive, selected experimental results for each of the work materials are tabulated in Table 3. From the experimental results, it can be seen that the surface roughness corresponding to each experiment soundly varies. This variation is also noticeable when the work material is changed in the same experimental conditions. Descriptive statistics for the experimental data associated with each alloy titanium alloy are presented in Table 4. Surface roughness pertaining to titanium, nickel, and aluminum alloy are represented as SR_TiA, SR_NiA, and SR_AlA, respectively. The roughness of the milled impression on titanium alloy (TiA) varied from 0.96 to 7.75 µm. Similarly, surface roughness of milled areas corresponding to nickel alloy (NiA) varies from a minimum of 2.16 and maximum of 5.67 µm. On the other end, the minimum value of roughness, in case of aluminum alloy (AlA), is found to be 0.96 µm and a maximum value of 5.51 µm. Such a huge difference indicates that there is a need for searching the most appropriate laser milling parameters to produce micro-cavities with minimum roughness. Therefore, the statistical models are developed and parametric optimization has also been carried out.

### 3.1. Parametric Effects on Surface Roughness

**Figure 2:**
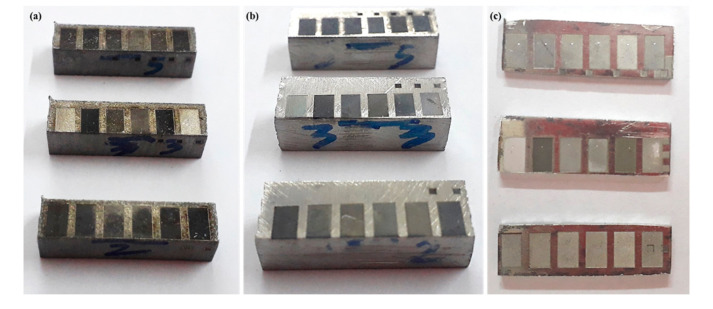
Work specimen after developing micro-depth impressions; (**a**) Inconel 718, (**b**) Ti 6Al 4V, and (**c**) Duralumin.

**Figure 3:**
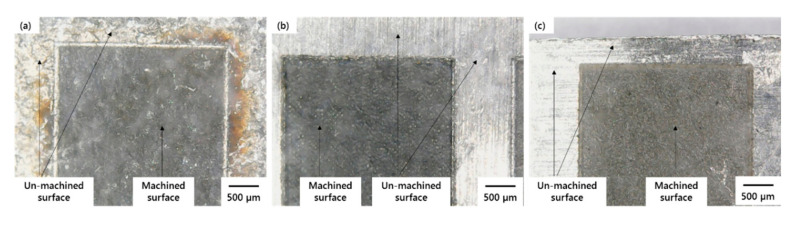
Microscopic enlarged views of laser-milled impression on: (**a**) TiA, (**b**) NiA, and (**c**) AlA.

A laser-milled impression on three substrate materials are presented in Figure 2. It can be witnessed that the cavities have significant color variation on each type of work sample. This coloration indicates that, by the change in laser parameters, not only is the roughness affected but also the surface color as well. The change in surface appearance is the result of oxidation occurring due to severe effects of laser parameters. Hence, impressions or cavities can be mainly classified into three categories: (1) dark or aggressive impressions with high oxidation, (2) moderate impressions with light color, and (3) bright impression with low oxidation. Figure 3 shows the enlarged microscopic views of laser-milled impression containing a high amount of oxidation.

**Figure 4:**
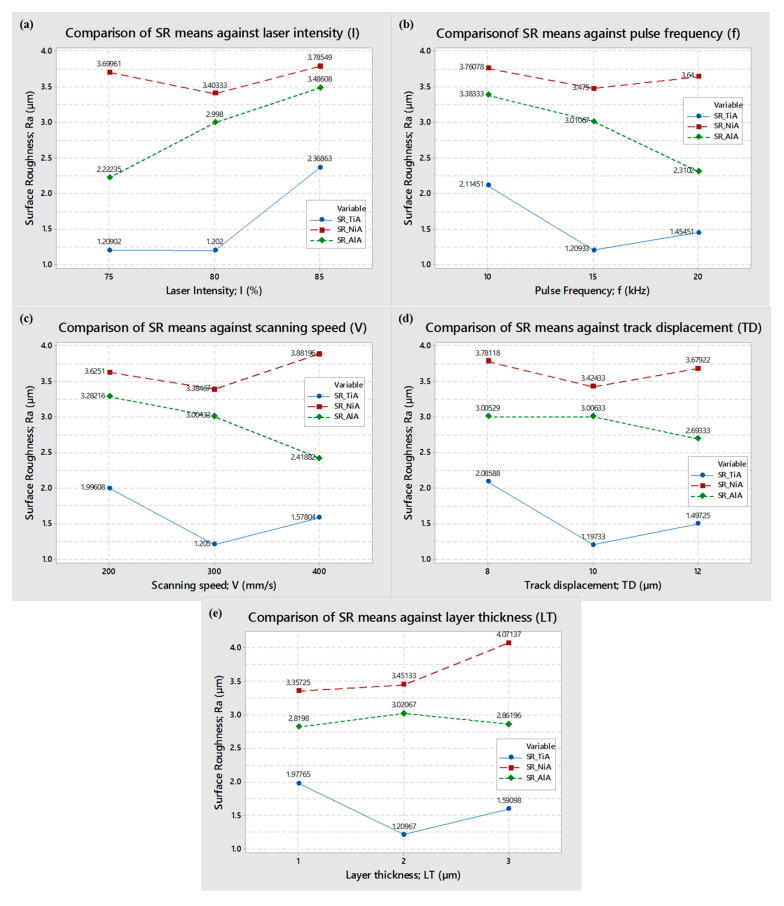
Mean effects of laser parameters on surface roughness of TiA, NiA, and AlA. (**a**) effect of current intensity, (**b**) effect of pulse frequency, (**c**) effect of scanning speed, (**d**) effect of track displacement, (**e**) effect of layer thickness.

In order to assess the effect of laser parameters over the surface roughness, the main effect plots and interaction effect plots are developed. Figure 4 shows the main effects of each of the five laser parameters for each of three alloys. As it can be seen from Figure 4a, with the increase in lamp current intensity, the roughness of the machined surface increased for titanium and aluminum alloy. However, the effect is not very prominent in case of nickel alloy. As the current intensity increased from 75% to 80%, there is no significant change in the roughness of TiA, but, beyond 80%, the roughness abruptly shoots up. The greater the current intensity, the more energy that is available for the material to get eroded, with the result being poor roughness. In the case of AlA, it can be seen that, with every incremental change in current, the roughness pattern follows a continuous increase indicating that, among the three alloys, aluminum alloy is more sensitive to current intensity. However, nickel alloy is relatively less responsive against the intensity level.

Figure 4b shows the effect of pulse repetition rate. For titanium alloy and aluminum alloy, the roughness follows an inverse relationship. The higher the frequency, the lower the roughness of the machined region. During each pulse on time, the material gets melted and, during the pulse off time, the molten metal gets removed, and this cycle continues through the pulse fluctuations.

Low pulse frequency means that the laser spot makes an interaction for a longer period and creates deeper craters. In this way, the resulting surface has a high amount of roughness. On the other hand, higher frequency leads to surface melting for a shorter period (due to shorter time lag between successive pulses) and results in shallow craters. Ultimately, a smooth surface is obtained. This phenomenon is relatively less significant in the case of NiA because of its higher melting point and low thermal conductivity (refer to Table 2) relative to aluminum alloy. Therefore, the effect of pulse frequency seems to be less influential for nickel alloy. However, this is not true in all the experimental runs since the graphical trends are based on average values of roughness against several experimental runs performed with each individual frequency level.

The effect of laser scan speed is shown in Figure 4c. The trends of mean values of roughness are similar to the trends observed in the case of pulse frequency. The primary concept behind the effect of scan speed is that the speed of laser scan allows the laser irradiations to interact with the substrate surface to cause melting, vaporization, and flushing of debris. Low scanning speed means that a high amount of time is available for the laser beam to melt and vaporize the molten debris. It causes excessive melting and deeper milling marks. The consequent surfaces have high roughness values. On the other side, higher scanning speeds allow the laser irradiation to interact for shorter periods of time for melting, and an insufficient material is removed. Incomplete melting under the action of higher scanning speeds also yields poor roughness. Comparing the mean values of surface roughness associated with each of the three alloys, the roughness is reduced when scanning speed changes from 200 mm/s to 300 mm/s. A further increase in scan speed deteriorates the milled surface, especially in the case of titanium and nickel alloys. The reason behind this deterioration is an insufficient melt pool caused by higher scan speed. Partial melting creates non-uniformity on the surface and high roughness. However, in the case of aluminum alloy (AlA), the highest level of scan speed is more favorable to achieve micro-cavities with minimum surface roughness.

The effect of pulse overlap or track displacement is graphically represented in Figure 4d. The concept behind the track displacement can be easily understood with the help of a schematic as shown in Figure 1. Lower value of track displacement allows the consecutive laser spots to be excessively overlapped. It indicates that the crater produced by the preceding laser spot is re-irradiated by the forthcoming laser spot and so on. Thus, every single preceding crater is melted under the action of multiple laser spots. As a result, the individual crater becomes deeper. Roughness of the milled region thus becomes high. On the other hand, if the track displacement is of higher value, the laser spots acquire less overlapping. The number of laser spots per unit area is low in case of high track displacement. It indicates that the neighboring spots are focusing on wider areas, and the resulting crates experience incomplete melting. Another reason behind this insufficient melting is the energy spread per laser spot. The amount of energy per spot is maximum at the spot center and gradually reduces outward from the spot center. Thus, the melting at micro-region is higher in the center of the individual crater and inefficient melting at the periphery of the single crater. In this way, the overall surface experiences irregularities and presents milling marks. It leads to poor roughness. This is why the trend lines of mean values of roughness acquire a v-shaped pattern especially for titanium and nickel alloys. Lower and higher levels of track displacements (8 µm and 12 µm) produced roughen micro-cavities, while the roughness of milled cavities is at its lower value when the track displacement is of 10 µm. However, for the case of duralumin (AA 2024), 12 µm overlapping produces smooth cavities.

The effect of layer thickness (*LT*) over the surface roughness is shown in Figure 4e. A mix of trend lines is found under the effect of changing layer thickness. The generalized phenomenon behind the setting of layer thickness is that the focus of the laser beam depends on the set value of *LT*. A value of 1 µm indicates that the laser beam is adjusted by 1 µm when a surface is completely scanned by the laser beam. In this way, there are 12 laser scans to complete a micro-impression of 12 µm depth. Similarly, a setting of *LT* = 2 µm results in six scanning layers to complete the targeted depth of impressions (12 µm depth)—whereas three layers or number of scans were executed under the setting of 3 µm layer thickness. If the actual thickness of the removed layer is equal to the theoretical thickness, then the laser focus occupies the same position for the next layer. However, if the milled layer is less than or greater than the desired layer thickness, then the focus of the laser beam doesn’t remain on the top surface of the upcoming layers. Due to the Gaussian mode of the laser beam, the utilization of energy density remains efficient if the focus is at the top surface. However, if the focus is above or below the top surface, the energy density available at the work surface is less utilized than the maximum energy. Thus, a difference in the thickness of actual removed layer and the theoretical layer thickness will cause inappropriate melting and the milled surface experiences different roughness. In the case of the nickel alloy, as the input value of the layer thickness is increased, poor roughness is achieved. At 3 µm thickness, the actual thickness of the removed material is less than 3 µm, and the adjusted focus during the next scanning cycle is lowered down by 3 µm. Thus, the new focus is inside the top surface instead of staying at the top surface. Eventually, the insufficient energy available at the top surface cause partial melting, and the result is high surface roughness. In the case of aluminum alloy, the mean values of roughness against each level of layer thickness are close to each other, thus any level of layer thickness may be suitable.

**Figure 5:**
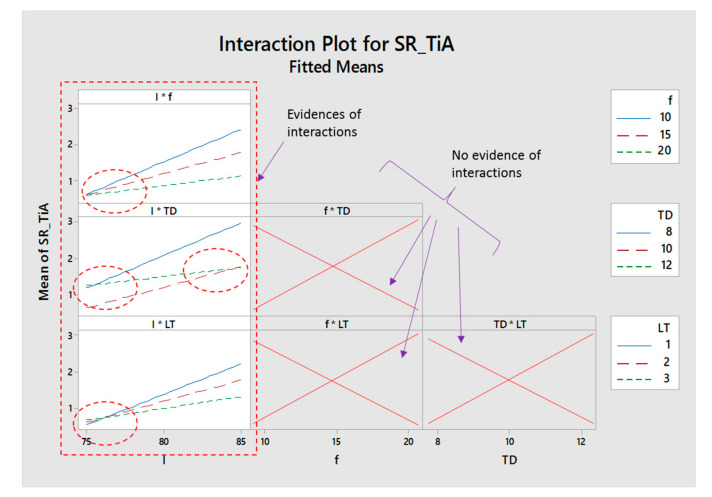
Interaction plot for titanium alloy.

**Figure 6:**
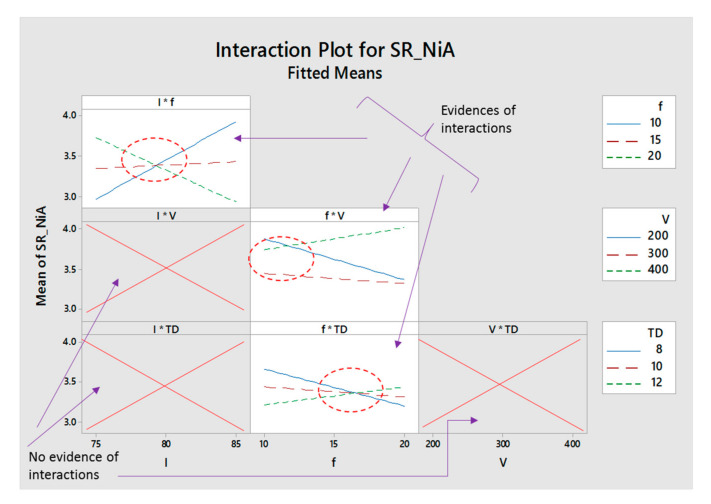
Interaction plot for nickel alloy.

**Figure 7:**
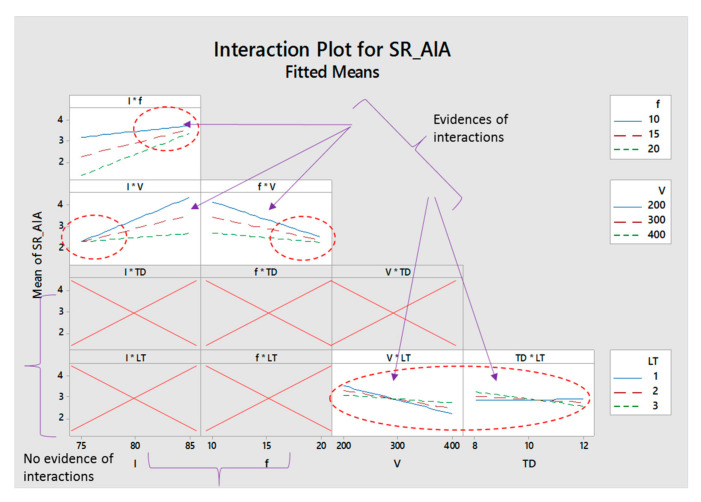
Interaction plot for aluminum alloy.

On the other hand, during the milling of titanium alloy, neither the lower level nor the upper level of layer thickness (1 µm and 3 µm) results in finished surface, as can be seen. It means that both the extreme levels imparts partial melting and the ultimate surface becomes rough. The lowest mean value of roughness is found to be 1.2 µm when a layer thickness of 2 µm is used for milling titanium alloy. The laser milling involves multiple factors, and it follows a complex machining phenomenon. In addition to linear effects of individual parameters, the milling performance is also a function of cross-dependency of different parameters. Therefore, interaction plots are also developed to access the cross-functionality of the laser parameters. Figure 5, Figure 6 and Figure 7 represent the interaction plots associated with titanium, nickel, and aluminum alloys, respectively.

Interactions of laser parameters for titanium alloy are shown in Figure 5. If the trend lines of two or more variables intersect with each other, it indicates the existence of an interaction effect—whereas, if there are parallel or non-intersecting lines, it means that the interaction of parameters doesn’t exist. In case of titanium alloy, three types of interaction can be found in Figure 5. Laser lamp current intensity (*I*) has significant interaction with pulse frequency (*f*), track displacement (*TD*), and layer thickness (*LT*), as represented by the dashed-rectangle. Inside each subset of the graph, dashed-elliptical callouts indicate that the trend lines of current intensity and trend lines of other parameters are crossing each other or are convergent at a particular level. Thus, from these interactions, it can be inferred that, while performing laser milling of titanium alloys, these parameters not only affect the milling performance in their individual capacity, but also they affect interactively. No evidence of interaction among other variables is found like interaction of pulse frequency with track displacement and layer thickness. Similarly, there is no interaction between track displacement and layer thickness.

Figure 6 shows the interaction plots for nickel alloy. It can be seen that evidence of interactions is available for only three variables. Lamp current intensity (*I*) strongly interacts with the pulse frequency (*f*) since all the three trend lines are mutually interactive with each other. It means that micro-milling through laser, lamp current, and pulse frequency not only affects the roughness by their individual values, but also they have a significant effect through their mutual interaction. Similarly, pulse frequency and scan speed (f*v), and pulse frequency and track displacement (f*TD) have an interactive effect during laser milling of nickel alloy. For the remaining combinations of laser parameters (such as I*V, I*TD, V*TD), no evidence is found for any interaction.

Five interaction effects are found in the case of aluminum alloy as can be seen from Figure 7. Lamp current intensity (*I*) creates an interaction effect with pulse frequency (I*f). Since the lines emerge at a high level of current intensity, the interaction between intensity and pulse frequency thus only come into existence when the current intensity is chosen at 85% or higher values. There is another interaction between lamp current and scanning speed (I*V) but at the lower level of current. It means that, while selecting the values of these two parameters, it should be considered that the effect on milling performance will also be prominent if a lower level of current intensity is taken. Similar interaction is observed between pulse frequency and scan speed (f*V), but the interaction is just at a pulse frequency of 20 kHz or above as the lines are converging at this level of pulse frequency. It is noticeable that, during laser milling of aluminum alloy, there is an extremely strong interaction between scan speed and layer thickness (V*LT), since the trend lines are intersecting and very congested. A similar kind of observation is for layer thickness and track displacement (LT*TD) as the response lines are crowded against each level of layer thickness and track displacement.

**Figure 8:**
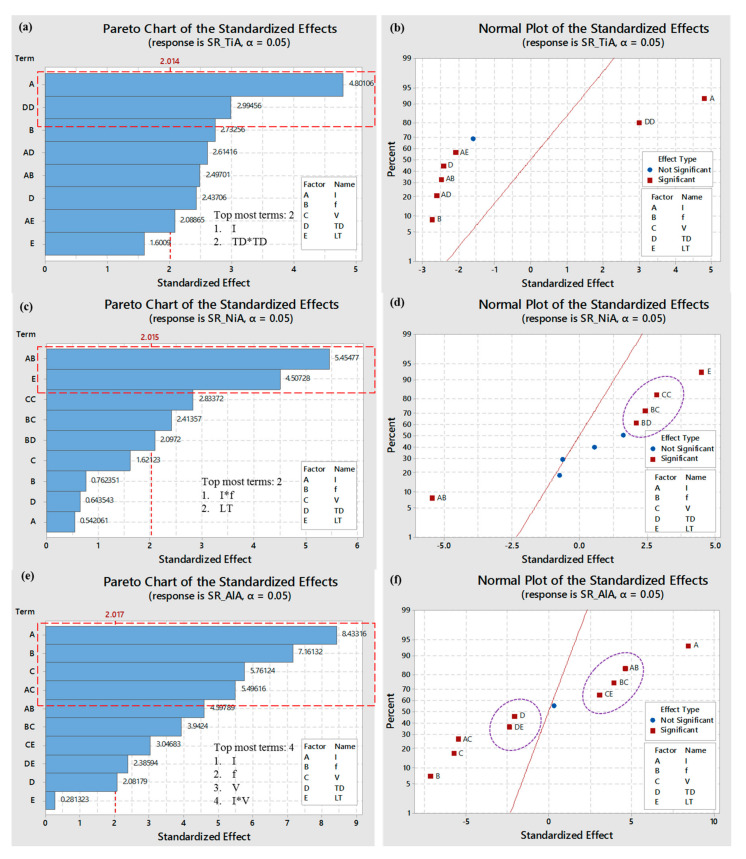
Standardized effects of laser parameters; (**a**) Pareto chart for TiA, (**b**) normal plot for TiA, (**c**) Pareto chart for NiA, (**d**) normal plot for NiA, (**e**) Pareto chart for AlA, and (**f**) normal plot for AlA.

After evaluating the mean effect and interaction effects, it can be concluded that the surface roughness of the micro- cavities produced through laser milling depends on various laser parameters. For each of the three alloys, this dependency varies differently. Therefore, in order to understand which factor significantly affects the response measure and which factor affects the roughness, the effect is statistically insignificant, and analysis of variance was conducted for each work material. Due to the conciseness, instead of detailed ANOVA results, a summary is presented in Table 5. Moreover, in order to access the strength as well as the direction of each effect, the Pareto and standardized effect analyses are performed. A summary of these analyses is included in Table 5, whereas the Pareto charts and normal plots of standardized effects are presented in Figure 8. Hence, the left half of Table 5 is the summary of ANOVA and the right half of the table is dedicated to a summary of standardized effects. Only significant terms are reported for ANOVA analysis. The criteria for significance is set at a 95% confidence interval with qualifying *p*-value of 0.05.

In case of TiA, a total of seven terms are found to be statistically significant affecting the roughness of micro- cavities. Among these seven terms, three terms are in their linear effects (*I, f,* and *TD*), one term is in its square form (TD*TD), whereas three terms in their interaction form (I*f, I*TD, and I*LT) are found to be significant. For the case of NiA, five terms qualified as significant terms including only one term (layer thickness; *LT*) in its linear effects. Likewise, only one term in its square form (V*V) and three interaction terms (I*f, f*V, and f*TD) are the significant terms. ANOVA analysis for AlA resulted in nine terms significantly affecting the roughness of milled cavity on aluminum alloy. Out of nine, four terms are in their linear effects (*I, f, V, TD*) and five terms are in their interaction effects (I*f, I*V, f*V, V*LT, and TD*LT).

To evaluate the strength of parametric effects, Pareto chart analysis is conducted. Within the Pareto analysis, the most significant terms are identified among the total number of significant terms. Moreover, the terms are categorized into two categories including largest effects and moderate effect. With the help of normal plots of standardized effects, terms with positive and negative effects are further segregated. The details of these number of parameters are shown in the second half of Table 5.

Figure 8 illustrates the graphical representation of these effects for easy understanding. The qualifying criteria for these two analyses are also based on a 95% confidence interval having α-value < 0.05. Normal effect plots are developed in such a way that the significant terms are spread around a neutral line. The terms falling on the right side of a neutral line indicates their positive effect, whereas the terms falling on the left side show their negative effect on the surface roughness. It must be noted that the data points or terms spreading close to the neutral line are those having a relatively weak effect, whereas the effect is assumed to be relatively stronger if the terms are spreading away from the center line. The strongest and weakest effect is independent of positivity and negativity criteria. Figure 8a,b are dedicated to titanium alloy (TiA). It can be seen that two parameters are the most influential ones as highlighted by a red-dashed rectangle. It includes the terms A and DD corresponding to lamp current intensity (I) and square effect of track displacement (TD*TD), respectively. These two terms fall on the right side of neutral as shown in Figure 8b. It means they have a positive effect—whereas the remaining five terms occupy the left place on a normal plot, indicating their negative effect on surface roughness. In the case of nickel alloy (NiA), five terms are significant and, among these, two terms are identified as having the largest effect. These are AB and E corresponding to interaction of pulse frequency with current intensity (I*f) and layer thickness (*LT*), as the bar charts of these two terms are the most prominent (refer to Figure 8c). The same observation is found in a normal plot (Figure 8d), wherein these two terms (AB and E) are far away from the reference line. The effect of E is positive, whereas the effect of AB is on the negative direction. The remaining three terms (CC, BD, and BC) are closer to each other (as shown by dotted-ellipse) and on the right side of reference line, indicating that the terms are significant, but, their effect is relatively not as strong as the other two terms (E and AB). With reference to aluminum alloy (AlA), it can be seen from Figure 8e that there are nine significant terms out of which four terms have the largest effect on surface roughness. These four terms include A, B, C, and AC, corresponding to current intensity (*I*), pulse frequency (*f*), scan speed (*V*), and interaction of current with scan speed (I*V), respectively. Based on their spread over the normal plot (Figure 8f), only one term (A) has a positive relationship, whereas the remaining three terms (B, C, and AC) have a negative effect.

Positive effect means that, with an increase in the value of that particular variable, the roughness of the micro- cavities also increase and vice versa. The parameters affecting the surface roughness but with weaker strength are highlighted with dashed-elliptical callouts.

### 3.2. Statistical Modeling and Validation

After performing the statistical analysis, the number of variables significantly affecting the surface roughness of each of the test alloys are identified. Later on, the strength and direction of each of these effects are discussed to understand the in-depth details of milling phenomenon with respect to machined surface roughness. It has been noticed that a variety of variables, several terms (linear, quadratic, and interaction), their strength, and direction of effect creates a very complicated picture. Moreover, descriptive statistics as discussed with reference to Table 4 indicates that there is huge variation in the roughness of the micro-impressions produced in three alloys (Ti 6Al 4V, Inconel 718, & AA 2024). Therefore, statistical models are developed so that the prediction of surface roughness can be made before performing the actual laser milling for micro- cavities on the mentioned alloys. The models associated with each of three alloys are presented in Equations (1)–(3). Only significant terms are included in these models and a stepwise procedure was followed to include the terms in each model:

(1) $$\begin{array}{ll} SR\_TiA=-39.6 & +0.732\left(I\right)+0.929\left(f\right)-0.51\left(TD\right)+3.97\left(LT\right)+0.1486\left(TD^{2}\right)\\ & -0.0124\left(I\times f\right)-0.0325\left(I\times TD\right)-0.052\left(I\times LT\right) \end{array}$$

(2) $$\begin{array}{ll} SR\_NiA=-11.68 & +0.276\left(I\right)+1.124\left(f\right)-0.026\left(V\right)-0.282\left(TD\right)+0.357\left(LT\right)\\ & +0.000037\left(V^{2}\right)-0.0178\left(I\times f\right)+0.0004\left(f\times V\right)\\ & +0.017\left(f\times TD\right) \end{array}$$

(3) $$\begin{array}{ll} SR_{NiA}=-4.58 & +0.168\left(I\right)-1.426\left(f\right)+0.0498\left(V\right)+0.106\left(TD\right)+0.236\left(LT\right)\\ & +0.0142\left(I\times f\right)-0.00085\left(I\times V\right)+0.0006\left(f\times V\right)\\ & +0.00235\left(V\times LT\right)-0.0921\left(TD\times LT\right) \end{array}$$

**Figure 9:**
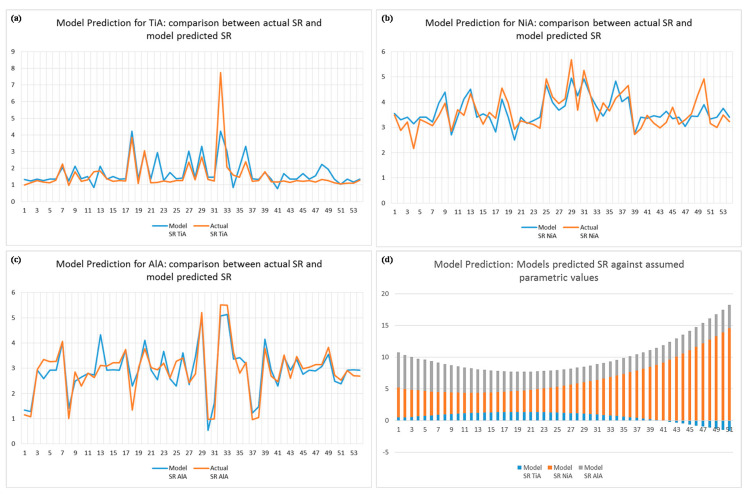
Verification of statistical models. Comparison of model predicted and actual experimental data for: (**a**) titanium alloy, (**b**) nickel alloy, (**c**) aluminum alloy, and (**d**) model predicted roughness using assumed ranges of laser parameters other than the used ranges.

The statistical models presented above are verified in two different ways. First, the model predicted values of surface roughness are compared with the actual experimental data set as shown in Figure 9a. It can be seen that the both of the data sets are very close to each other since each of the trend lines associated with each of the three alloys superimpose each other. This indicates that the model prediction is well-estimated compared to actual experimental values. Secondly, the models are further verified by re-dining the range of each laser parameter with an assumed set of values. Since the presented study is carried out with the parameters’ levels chosen after extensive trials, in order to validate the models, a new range of each parameter is defined. The new range starts from far beyond the lowest and highest range used for actual experiments. For example, the actual range of laser lamp current intensity was 75–85% with three levels (75, 80, and 85) having an increment of 5—while the assumed range of current intensity is taken as 70–95% with 51 levels having an increment of 0.5. Similarly, an actual range of scan speed was taken as 200–400 mm/s having an increment of 100 mm/s (levels: 200, 300, and 400 mm/s). For model verification, the range is selected within 100–850 mm/s having an increment of 15 mm/s. The same is the case for the other three variables. In this way, a total of 51 predicted solutions are estimated using each proposed model. The results are graphically presented in Figure 9b. The graph reveals that the minimum surface roughness obtained through solution prediction falls in the middle of the graph. It indicates that the minimum surface roughness can only be obtained if the range of each laser parameter falls in the middle of the newly assumed range. It also verifies that the actual selected range of each laser parameter was well-defined after trial experiments. Graphs show that the laser parameters having values below the lower level yield high surface roughness as well as the parameters with values higher than the upper level of each parameter also produce high surface roughness. Thus, it can be inferred that the models are so robust that the laser operator can use these models to predict the surface roughness in each of the three alloys (NiA, TiA, and AlA) prior to performing an extensive set of trials. There are several applications wherein high surface roughness is desired, for instance in biomedical implants of titanium alloys. Thus, if the operator wants to produce micro-impressions with any desired level of surface roughness (other than minimum surface roughness), the models can effectively be utilized to identify the appropriate parametric settings and the resulting roughness of the laser milled surfaces.

### 3.3. Optimality Search and Validation

**Figure 10:**
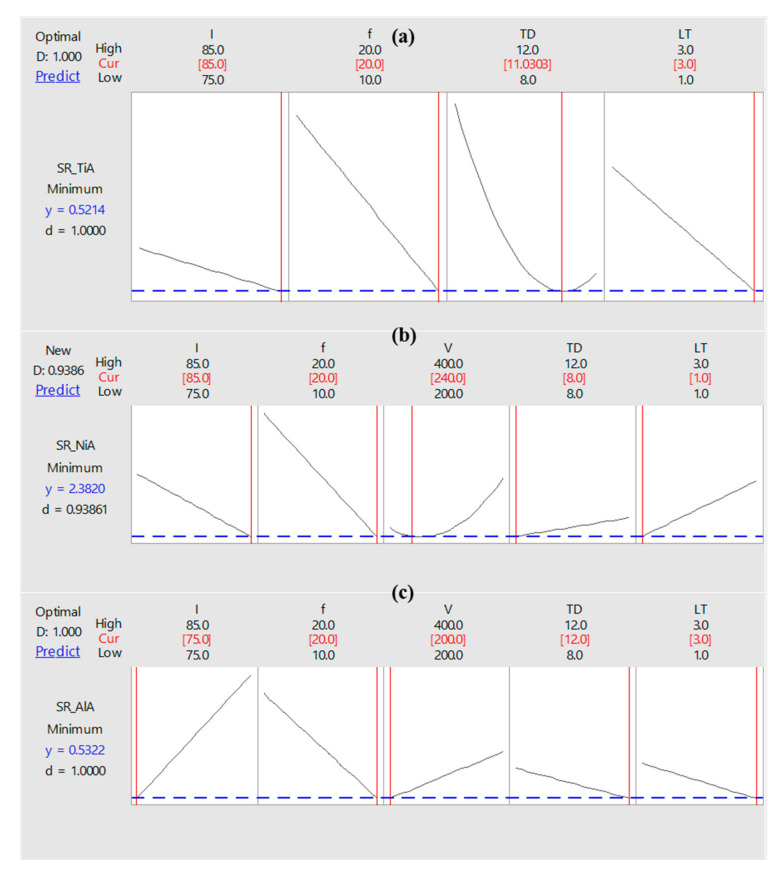
Optimization plot for surface roughness in: (**a**) TiA, (**b**) NiA, and (**c**) AlA.

In most of the engineering applications, minimum surface roughness is desired. Therefore, for each laser machining community, the optimized set of laser parameters can help to directly use the laser milling process especially for generating micro-impressions. Hence, optimized laser parameters are proposed in this study as well. The criteria for optimization is set as “minimum roughness” with a weight and importance of unity (refer to Table 6). The optimization plot is shown in Figure 10. The top three lines in each of the sub-plots of Figure 10 represent the values of laser parameters. The red-colored values indicate the optimized laser parameters giving minimum roughness in each of the three cases (TiA, NiA, AlA). On the *y*-axis, the minimum roughness values are indicated with blue numbers. It shows that, by the use of optimized laser parameters, micro- cavities on titanium alloy (TiA), nickel alloy (NiA), and aluminum alloy (AlA) may have surface roughness of 0.52 µm, 2.38 µm, and 0.53 µm. Looking at the desirability values, it can be seen that the desirability in case of TiA, NiA, and AlA is 1.0, 0.93, and 1.0, respectively. The set of optimized laser parameters for each of the three alloys are presented in Table 7 including the fitted values, standard error fit, and confidence interval of the surface roughness.

To further authenticate the proposed optimized laser parameters, confirmatory experiments have also performed. Firstly, multiple response prediction was carried out through optimality search. Three prediction results for each of three alloys are presented in Table 8.

**Figure 11:**
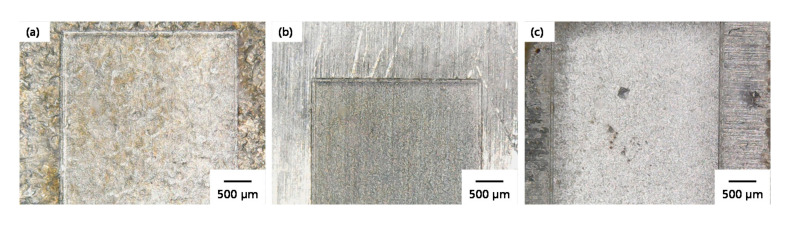
Micro-impressions produced under the optimized laser parameters on; (**a**) TiA, (**b**) NiA, (**c**) AlA.

Every first solution was considered as the best optimized solution. Thus, the confirmatory experiments were performed using laser parameters mentioned in Table 8. The micro-impressions produced under the utilization of an optimized set of parameters are shown in Figure 11. The micro-impressions were found to very dark when produced with non-optimized laser parameters (see Figure 2), while it can be seen that these cavities are also very bright in color indicating the absence or very low amount of oxidation. The measurement of surface roughness was again performed three times on different regions of micro- cavities and the average value was compared with the fitted values. The difference in the actual roughness values and the fitted values was found to be less than 10%. For example, the fitted values of surface roughness for TiA, NiA, and AlA are 0.52 µm, 2.37 µm, and 0.53 µm, whereas the actual surface roughness was 0.56 µm, 2.46 µm, and 0.54 µm, respectively.

## 4. Conclusions

Through laser milling, micro-impressions of 12 µm depth in have been produced on three different alloys (titanium alloy; Ti 6Al 4V, nickel alloy; Inconel 718, and aluminum alloy; AA 2024) using a response surface methodology based design for the experiment. The effect of five laser parameters including lamp current intensity (*I*), pulse frequency (*f*), scanning speed (*V*), layer thickness (*LT*), and track displacement (*TD*) on surface roughness of the milled cavities have been evaluated though various statistical analyses. Statistical models are developed to predict the roughness prior to actual machining and optimized laser parameters are sought to yield micro- cavities with minimum surface roughness. Based on the results and discussion, the following list of conclusions may be inferred:

i. Micro-impressions on titanium, nickel, and aluminum alloys can be produced through laser milling. However, under the non-optimized laser parameters, the milled surfaces experience excessive oxidation and result in dark impressions with a high degree of surface roughness.
ii. Among the laser parameters, the behavior of each laser parameter, with respect to three substrate materials, is almost similar in nature but differs in its strength. Each substrate gets affected differently under laser parameters. Aluminum alloy is found to be more sensitive compared with the other two alloys (TiA and NiA).
iii. In case of titanium alloy, seven terms (*I*, *TD^2^*, I*f, I*TD, I*LT) are found to be significantly affecting the roughness of milled surfaces (refer to Table 5). The most significant terms include lamp current intensity *(I)* and track displacement in its quadratic nature (*TD^2^*).
iv. In the case of nickel alloy, only five terms (LT, V^2^, I*f, f*V, and f*TD) significantly affect the milling performance. The term LT corresponding to layer thickness has the largest positive effect.
v. Aluminum alloy is relatively sensitive to the laser parameters and nine terms (I, f, V, TD, I*f, I*V, f*V, V*LT, and TD*LT) significantly affect the surface roughness of the micro-impressions. However, among these nine terms, the roughness is largely affected by the laser lamp current intensity (*I*).
vi. Statistical models are validated and can be effectively used to predict the roughness prior to produce micro- cavities. Since several engineering applications (e.g., biomedical implants) need to have surfaces with a desired level of roughness, the models can also be used to identify the suitable level of laser parameters to achieve a desired level of surface roughness.
vii. Optimized parameters to produce micro-cavities with minimum surface roughness include (refer to Table 8, solution 1):
  - For titanium alloy: Current intensity (I) at 85%, pulse frequency (f) at 20 kHz, laser scan speed (V) at 250 mm/s, track displacement (TD) at 11 µm, and layer thickness (LT) at 3 µm,
  - For nickel alloy: Current intensity (I) at 85%, pulse frequency (f) at 20 kHz, laser scan speed (V) at 256.5 mm/s, track displacement (TD) at 8 µm, and layer thickness (LT) at 1 µm,
  - For aluminum alloy: Current intensity (I) at 75%, pulse frequency (f) at 20 kHz, laser scan speed (V) at 200 mm/s, track displacement (TD) at 12 µm, and layer thickness (LT) at 3 µm,
viii. Micro-impressions produced under the optimized set of laser parameters have surface roughness of 0.56 µm, 2.46 µm, and 0.54 µm, on titanium alloy, nickel alloy, and aluminum alloy, respectively. The milled impressions on each of the three alloys have bright surface appearance, if optimized parameters are used for milling.

## Figures and Tables

**Table 1:** Important properties of work materials.

Property	Ti-6Al-4V [55,56,57]		AA 2024 [58,59,60]		Inconel 718 [61,62,63]	
	Condition	Value	Condition	Value	Condition	Value
**Melting Temperature** **°C**	-	1604–1660	-	520	-	1260–1335
**Thermal Conductivity** **W/m** **°C**	*K* = 0.0156.T + 7	*T ≤ T_m_:* *K* = 32.74	20< *T* <300: *K* = 0.247.*T* + 114.4	164–220 [58] 121.8–176.4 [60]	23–1200 °C	10.6–29.6
**Emissivity**	132–760 °C >760–1100 °C	0.22–0.3 0.7–0.98	600–800 *K*	0.1–0.3 [64]	100-200 °C 650–1300 *K*	0.24–0.33 [65] 0.2–0.7 [62]
**Absorptivity @ 1.064 µm Wavelength**	500–1400 °C 928 °C	0.28–0.41 [55] 0.38 [57]	-	0.07 [66]	300–1700 *K*	0.1–0.55 [61]

**Table 2:** Details of variables (symbols, units, levels) and responses.

Variable	Unit	Levels			Response	Unit
Lamp current intensity (*I*)	A (%)	75	80	85	Surface roughness (SR_TiA)	R_a_ (µm)
Pulse frequency (*f*)	kHz	10	15	20	Surface roughness (SR_NiA)	R_a_ (µm)
Scanning speed (*V*)	mm/s	200	300	400	Surface roughness (SR_AlA)	R_a_ (µm)
Track displacement (*TD*)	µm	8	10	12		
Layer thickness (*LT*)	µm	1	2	3		

**Table 3:** Response surface methodology (RSM) with face centered central composite design (FCCCD) and the selected experimental results after laser beam machining (LBM) of titanium, nickel, and aluminum alloy.

Run #	Parameters					Responses											
	*I* (%)	*f* (kHz)	*V* (mm/s)	*TD* (µm)	*LT* (µm/scan)	SR_TiA (µm)				SR_NiA (µm)				SR_AlA (µm)			
						R_a1_	R_a2_	R_a3_	Avg. R_a_	R_a1_	R_a2_	R_a3_	Avg. R_a_	R_a1_	R_a2_	R_a3_	Avg. R_a_
**1**	75	20	200	12	1	1.06	0.98	0.96	1.00	3.5	3.48	3.5	3.49	1.06	1.2	1.2	1.15
**2**	75	20	200	8	1	1.12	1.16	1.12	1.13	3.1	2.92	2.62	2.88	1.14	0.98	1.12	1.08
**3**	80	15	300	10	2	1.26	1.38	1.18	1.27	3.74	3.46	2.44	3.21	2.92	2.86	3.08	2.95
**4**	75	10	400	8	1	1.14	1.2	1.18	1.17	2.16	2.12	2.22	2.17	3.08	3.36	3.6	3.35
**5**	80	15	300	10	2	1.14	1.1	1.18	1.14	3.6	3.66	2.7	3.32	3.26	3.2	3.3	3.25
**6**	80	15	300	10	2	1.24	1.3	1.3	1.28	3.1	3.12	3.4	3.21	3.42	3.18	3.22	3.27
**7**	85	20	200	8	3	2.38	2.22	2.18	2.26	3.08	3.16	2.98	3.07	3.9	4.08	4.2	4.06
**-**	-	-	-	-	-	-	-	-	-	-	-	-	-	-	-	-	-
**-**	-	-	-	-	-	-	-	-	-	-	-	-	-	-	-	-	-
**21**	80	15	300	10	2	1.08	1.16	1.14	1.13	2.92	3.44	3.42	3.26	2.74	3.16	3.2	3.03
**22**	85	20	400	8	1	1.26	1.1	1.08	1.15	3.26	3.28	3.02	3.19	3	2.94	2.88	2.94
**23**	75	10	200	8	1	1.26	1.22	1.22	1.23	3.22	3.06	3.06	3.11	3.38	3.24	3	3.21
**24**	85	20	400	12	1	1.14	1.2	1.2	1.18	2.82	3	3.1	2.97	2.6	2.72	2.54	2.62
**25**	75	20	400	8	3	1.18	1.28	1.3	1.25	4.76	4.92	5.1	4.93	3.5	3.14	3.18	3.27
**26**	75	10	200	8	3	1.24	1.24	1.3	1.26	4.28	4.22	4.1	4.20	3.54	3.34	3.32	3.40
**27**	85	10	400	12	1	2.38	2.36	2.36	2.37	3.98	3.94	3.92	3.95	2.78	2.16	2.34	2.43
**-**	-	-	-	-	-	-	-	-	-	-	-	-	-	-	-	-	-
**-**	-	-	-	-	-	-	-	-	-	-	-	-	-	-	-	-	-
**41**	75	15	300	10	2	1.1	1.22	1.22	1.18	3.52	3.44	3.48	3.48	2.42	2.56	2.44	2.47
**42**	80	10	300	10	2	1.26	1.2	1.26	1.24	3.18	3.12	3.2	3.17	3.68	3.48	3.4	3.52
**43**	80	15	300	10	2	1.14	1.18	1.16	1.16	3.04	3.04	2.86	2.98	2.58	2.6	2.62	2.60
**44**	80	15	200	10	2	1.2	1.34	1.26	1.27	3.32	3.18	3.1	3.20	3.36	3.48	3.54	3.46
**45**	80	15	300	12	2	1.28	1.18	1.2	1.22	4.08	3.38	3.94	3.80	3.02	2.92	3.02	2.99
**46**	80	15	300	10	2	1.22	1.26	1.28	1.25	2.94	3.18	3.26	3.13	3.04	3.04	3.04	3.04
**47**	80	15	300	10	1	1.18	1.2	1.16	1.18	3.32	3.32	3.26	3.30	3.16	3.14	3.12	3.14
**48**	80	15	300	8	2	1.36	1.26	1.34	1.32	3.46	3.6	3.54	3.53	3.14	3.18	3.12	3.15

**Table 4:** Descriptive statistics: SR_TiA, SR_NiA, SR_AlA.

Variable	Mean	SE Mean	StDev	Minimum	Maximum	Skewness	Kurtosis
**SR_TiA**	1.57	0.13	1.01	0.96	7.75	4.70	26.44
**SR_NiA**	3.61	0.09	0.68	2.16	5.67	0.88	0.83
**SR_AlA**	2.90	0.14	1.01	0.96	5.51	0.04	1.2

**Table 5:** ANOVA summary for surface roughness.

Substrate Material	ANOVA Summary					Pareto and Normal Effects Summary			
	Significant Terms			Total Significant Terms	Most Significant Terms	Largest Effects		Moderate Effects	
	Linear Terms	Square Terms	Interaction Terms			Largest +ve Effect	Largest −ve Effect	Moderate +ve Effect	Moderate −ve Effect
**TiA**	3 terms *1*. I *2*. f *3*. TD	1 term *1*. TD*TD	3 terms *1*. I*f *2*. I*TD *3*. I*LT	7 terms	2 terms *1*. I *2*. TD*TD	1 term *1*. I	4 terms *1*. f *2*. I*TD *3*. I*f *4*. TD	1 term *1*. TD*TD	1 term *1*. I*LT
**NiA**	1 term *1*. LT	1 term *1*. V*V	3 terms *1*. I*f *2*. f*V *3*. f*TD	5 terms	2 terms *1*. I*f *2*. LT	1 term *1*. LT	1 term *1*. I*f	3 terms *1*. V*V *2*. f*V *3*. f*TD	0 terms
**AlA**	4 terms *1*. I *2*. f *3*. V *4*. TD	0 terms	5 terms *1*. I*f *2*. I*V *3*. f*V *4*. V*LT *5*. TD*LT	9 terms	4 terms *1*. I *2*. f *3*. V *4*. I*V	1 term *1*. I	3 terms *1*. f *2*. V *3*. I*V	3 terms *1*. I*f *2*. f*V *3*. V*LT	2 terms *1*. TD *2.*. TD*LT

**Table 6:** Optimization goal for TiA, NiA, and AlA.

Response	Goal	Target	Upper	Weight	Importance
**SR_TiA**	Minimum	0.96	7.75	1	1
**SR_NiA**	Minimum	2.16	5.67	1	1
**SR_AlA**	Minimum	0.96	5.51	1	1

**Table 7:** Multiple response optimal settings and optimization results for TiA, NiA, and AlA.

Variable	Optimal Settings			Optimization Results			
	TiA	NiA	AlA	Response	Fit	SE Fit	95% CI
***I***	85.0	85.0	75.0	SR_TiA	0.521	0.312	(0.106, 1.149)
***f***	20.0	20.0	20.0	SR_NiA	2.373	0.219	(1.931, 2.815)
***V***	300.0	240.0	200.0	SR_AlA	0.532	0.248	(0.032, 1.032)
***TD***	11.03	8.0	12.0				
***LT***	3.0	1.0	3.0				

**Table 8:** Multiple response prediction solutions for TiA, NiA, and AlA.

Response	Solution	*I* (%)	*f* (kHz)	*V* (mm/s)	*TD* (µm)	*LT* (µm)	SR Fit (µm)	Composite Desirability
**SR_TiA**	1	85.0	20.0	250	11.03	3.00	0.52	1.000
	2	80.0	15.0	250	10.51	3.00	0.96	0.999
	3	75.0	20.0	250	11.69	1.00	0.98	0.997
**SR_NiA**	1	85.0	20.0	256.5	8.0	1.00	2.37	0.941
	2	85.0	20.0	233.5	8.0	1.00	2.39	0.935
	3	85.0	20.0	245.2	8.14	1.02	2.39	0.935
**SR_AlA**	1	75.0	20.0	200.0	12.0	3.00	0.53	1.000
	2	75.0	20.0	200.0	9.44	3.00	0.96	1.000
	3	75.0	20.0	200.0	8.00	1.00	1.27	0.932
